# Proline‐rich polypeptides (Colostrinin^®^/COLOCO ^®^) modulate BDNF concentration in blood affecting cognitive function in adults: A double‐blind randomized placebo‐controlled study

**DOI:** 10.1002/fsn3.3187

**Published:** 2023-01-11

**Authors:** Hanna Banasiak‐Cieślar, Dawid Wiener, Magdalena Kuszczyk, Katarzyna Dobrzyńska, Antoni Polanowski

**Affiliations:** ^1^ Geo‐Poland sp. z o. o Kutno Poland; ^2^ Department of Design (School of Form) SWPS University of Social Sciences and Humanities Warsaw Poland; ^3^ Department of Animal Products Technology and Quality Management University of Environmental and Life Sciences Wroclaw Poland

**Keywords:** BDNF, CANTAB, cognitive function, COLOCO^®^, Colostrinin^®^, dementia, neurotrophins, proline‐rich polypeptides

## Abstract

Proline‐rich polypeptides (PRPs complex also known as COLOCO^®^, Colostrinin^®^) consist of low‐molecular weight peptides ranging up to 10 kDa, isolated from the bovine colostrum obtained up to 48 h postpartum. PRPs have been shown to affect processes involved in inflammation, brain aging, and neurodegeneration. The aim of this study was to investigate the effect of Colostrinin^®^ (COLOCO^®^) on the cognitive abilities of healthy volunteers in three different age groups using the CANTAB tool in a double‐blind randomized placebo‐controlled study. BDNF serum level was used as a physicochemical marker of improvement of the cognitive skills. Three hundred and sixty‐one healthy volunteers were divided into three study groups aged 18–24, 25–54, and 55–75; each group was then divided into two subgroups which took either placebo or tested lozenge with 120 μg of PRPs for the period of 4 months. The CANTAB battery test was used to measure the efficacy of PRP in the context of cognitive functioning. After the treatment with COLOCO^®^, we observed differences within MoCA score in the oldest patients, improvement in DMS and drop in PAL scores within the youngest group, drop in RTI and improvement in RVP scores within the middle‐aged group. It was observed that serum BDNF level increased in all study groups which confirms cognitive improvement. In conclusion, we have shown that Colostrinin^®^ exhibits cognitive enhancing effects, probably through the modulation of BDNF concentrations.

## Graphical abstract

Colostrinin^®^, isolated from the bovine colostrum, has been shown to affect processes involved in inflammation, brain aging, and neurodegeneration. The aim of this study was to investigate the effect of colostrinin taking daily on the cognitive abilities of healthy volunteers in a double‐blind randomized placebo‐controlled study. We observed differences and improvement within results with cognitive test abilities. As a confirmation of cognitive abilities' enhancement, it was observed that serum BDNF level increased in all study groups, which confirms cognitive improvement.

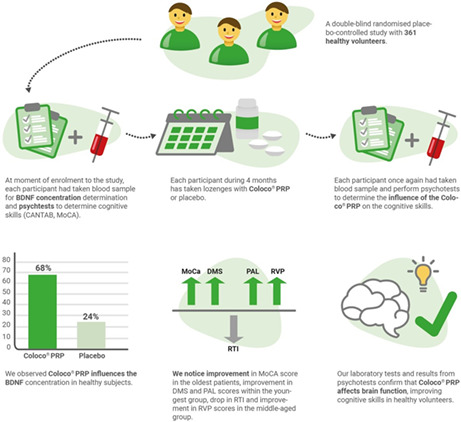

## 1 INTRODUCTION

Colostral proline‐rich polypeptides complex (PRPs) is low‐molecular weight complex of peptides ranging up to 10 kDa, isolated from the bovine (COLOCO^®^, Colostrinin^®^) or ovine colostrum obtained up to 48 hours postpartum. In vitro and in vivo studies demonstrated that colostrinin is an immunologically active agent that keeps a balance between pro‐ and anti‐inflammatory processes (Lommatzsch et al., 2005). The analogs of the PRPs were identified in the other types of mammalian colostrum, suggesting their crucial role in infant development. Unique PRP complex is of particular interest to researchers, since PRPs were shown to affect the processes that scientific research has identified as potential therapeutic targets for modulating upstream events associated with the inflammation, brain aging, and neurodegeneration, and exhibits promising effects in Alzheimer's disease (AD) (Popik et al., 1999; Zabłocka et al., 2007; Zabłocka et al., 2020).

### 1.1 COLOCO^®^ (Colostrinin^®^)

COLOCO^®^ (Colostrinin^®^) is an isolate extracted from the first milk of cows—colostrum obtained by patented technology. Proline content in COLOCO^®^ is about 22%. COLOCO^®^ is neither glycosylated nor phosphorylated. More than a half (51% of the total) of protein contains hydrophobic amino acids (Kruzel et al., 2001). Due to its high proline content, has a beneficial effect on memory, concentration, cognitive processes, logical thinking ability, improves the speed of reaction and association of facts. It has exceptional anti‐inflammatory and antioxidant properties; regulates the growth, maturation, and differentiation of lymphocytes; inhibits the accumulation of toxic proteins. Animal studies have shown that Colostrinin^®^ improves memory, delays the onset and progression of dementia and long‐term memory loss. (Lemieszewska et al., 2016)

The low‐molecular weight of COLOCO^®^ allows this bioactive substance to penetrate through cell membranes. It was demonstrated that colostrinin is a mixture of at least 32 peptides ranging in size from 0.5 to 3 kDa (Rattray, 2005). Moreover, Colostrinin^®^ influences the integration of blood vessel walls and increases their permeability. (Wieczorek et al., 1979) Colostrinin is bioavailable in humans after oral intake of 50–200 μg, according to studies (Leszek et al., 1999). Colostrinin contained in milk of mammals dilates blood vessels to facilitate the transport of precious substances contained in colostrum. In studies on vascular permeability, scientists have shown that COLOCO^®^ affects the release of vasoactive factors—dilating blood vessels in the gastrointestinal tract. (Wieczorek et al., 1979)

### 1.2 Brain‐derived neurotrophic factor

Brain‐derived neurotrophic factor (BDNF) is one of neuronal growth factors that stimulates and controls neurogenesis and promotes neuronal survival (Acheson et al., 1995). This protein belongs to the family of neurotrophins—peptides that support the function of the nervous system by influencing the development and maturation of neurons (Arancio & Chao, 2007; Bath et al., 2012). Neurodegenerative and neuropsychiatric diseases may be induced by defects in synaptic plasticity due to insufficient expression of BDNF and other neurotrophic factors. Neurotrophins prevent the premature death of neurological cells, accelerate the proliferation and maturation of nerve cells, and are therefore crucial for the processes of neurodegeneration. They are involved of pathophysiology of neurodegenerations, especially Parkinson's disease and Alzheimer's disease. It is therefore important to ensure optimal levels of neurotrophins such as BDNF as a preventive measure for dementia, neuropsychiatric and neurodegenerative diseases (Marie et al., 2018; Palasz et al., 2020). Moreover, BDNF influences learning processes by regulating the process of synaptogenesis and is involved in neuronal communication and the proper brain activity. BDNF ensures the stability of synapses, thereby strengthening the connections formed between neurons and ensuring long‐term memory (Bekinschtein et al., 2008). In healthy humans, BDNF both in the brain and circulating in the bloodstream increases its concentration with age. The highest level is observed in the third decade of life, and then, it slowly gradually decreases (Tapia‐Arancibia et al., 2008).

The Cambridge Neuropsychological Test Automated Battery (CANTAB) software was created to evaluate cognitive deficiencies in patients with neurodegenerative disorders or brain injuries. Although the test battery was designed and developed for patients with diagnosed cognitive impairments, scientific reports exhibit the usage of those to assess cognitive outcomes within healthy individuals (Chew et al., 2015; Danthiir et al., 2011; Power et al., 2022). The test allows evaluating memory skills, attention, and executive functions of patients. The test can be conducted on the touch screen and its results allow assessment of the cognitive functions. It permits the dissection of complicated tasks typically utilized in clinical evaluation into their cognitive components (Fray & Robbins, 1996).

The aim of the proposed study was to investigate the effects of COLOCO^®^ (Colostrinin^®^) on the cognitive abilities of healthy volunteers in three different age groups using the CANTAB tool. As a physicochemical marker of improvement of the cognitive skills, BDNF serum level was used.

## 2 MATERIAL & METHODS

The randomized double‐blind study was approved (approval number: 35/13; KB/901/13) by the Bioethics Committee of the District Medical Chamber in Warsaw, Poland. The study included 361 healthy Caucasian volunteers with normal fine motor skill, vision, and hearing. Informed consent was obtained from each participant prior to their inclusion in the study.

Drug use, alcohol dependence, mood disorders, psychotic disorders, and diagnosis of serious mental illness were used as exclusion criteria. Detailed guidelines for the inclusion and exclusion criteria of volunteers to the study are presented in Table 1.

**TABLE 1:** Inclusion and exclusion criteria of enrolment to the study.

Inclusion criteria	Exclusion criteria
Age MT group: ≥18 and < 25 ADT group: ≥25 and < 55 AAMI group: ≥55 and ≤ 75	Medical history of Major depressive disorder (or current episode under treatment) Schizophrenia Other psychotic disorders, bipolar disorder (past 5 years), or substance (including alcohol) related disorder (past 2 years)
Scores to meet specific target groups criteria MT = 5 in 5 questions screening questionnaire ADT ≥3 in 6 point Adult ADHD Self‐Report Scale AAMI <26 points in Montreal Cognitive Assessment scale	Clinical evidence or history of any of the following within specified period before Baseline Cerebrovascular accident (past 2 years) Transient ischemic attack (past 6 month) Significant head injury with associated loss of consciousness, skull fracture, or persisting cognitive impairment
Being able to comply with the study procedures in the view of investigator	Diagnosed with significant CNS disease: Alzheimer's disease, Lewy body dementia, Parkinson's disease, multiple sclerosis, progressive supranuclear palsy, hydrocephalus, Huntington's disease, epilepsy (even after single prior seizure)
Participant is able to read, understand, and provide written informed consent	Abnormal serum chemistry laboratory value at baseline deemed to be clinically relevant by the investigator

### 2.1 COLOCO ^®^ (Colostrinin^®^) isolation

COLOCO^®^ isolation technique is protected by a patent UPRP No. 218693. The liquid cows' colostrum is cooled and centrifuged for 20 minutes to separate the fat, then diluted with EDTA solution and left for 30 minutes. The mixture is then cooled, and cooled acetone is added and the mixture is extracted with stirring. The mixture is clarified at reduced temperature, optionally anion acetate is added to the supernatant to form a two‐phase system and the water–protein phase is separated from the acetone phase. The water–protein phase of the supernatant is then heated under reduced pressure to remove acetone, acidified and filtered through a 1000 Da membrane. The retentate is optionally concentrated and/or dried to give a peptide preparation with about 20% proline and about 18% acidic amino acids. (Details PAT ‐ P.388408, n.d.)

### 2.2 Study groups

Three hundred and sixty‐one healthy volunteers recruited in two research centers (Poznan and Warsaw) have been divided into three groups: multitaskers (MT)—122 adults of both sexes in the age between 18 and 25; attention‐deficit trait (ADT)—116 adults of both sexes in the age between 26 and 55; age‐associated memory impairment group (AAMI/OLD)—123 adults of both genders above 55 years old. Each participant fulfilled a questionnaire at enrolment to the study. The enrolment questionnaire was used to minimalize the impact of factors positively or negatively affecting BDNF concentration. Each study group was divided into two subgroups that took either a placebo or tested lozenge with Colostrinin^®^ (COLOCO^®^). Lozenges were taken by the subjects at a daily dose of 120 μg for 4 months, with a 14‐day break after each 30‐day cycle of use. Pulse regimen with 2 weeks of hiatus was ordered due to the tachyphylaxis for the active ingredient described in the literature (Leszek et al., 1999). This study exhibits strict tachyphylaxis to colostrinin after 3 weeks of oral administration within healthy individuals. To avoid such a phenomenon, we decided to order “washout” period also at our study groups.

### 2.3 Blood samples

Ten milliliters of venous blood was sampled at the baseline during first visit and third visit (at the beginning and at the end of the study 16 weeks thereafter). Venous blood was withdrawn into EDTA containing tubes between 7.30 and 9.30 a.m. to minimize effects of a circadian rhythm on BDNF concentrations. Blood for serum samples was subsequently centrifuged (10 min; 3000 rpm; room temperature) and frozen at −20°C until further analysis.

### 2.4 Enzyme‐linked immunosorbent assay (ELISA)

The BDNF analysis was conducted using a commercially available ELISA kit (Human BDNF DuoSet ELISA, R&D Systems) according to the manufacturer's protocol. Plates were blocked for 2 hours in reagent diluents (1% BSA/PBS). Serum samples for BDNF were run undiluted. Plates were incubated with 100 μl of serum samples overnight at 4°C. All samples and standards were run in duplicates. The optical density was measured using a FLUOstar Omega microplate reader (BMG Labtech, Ortenberg, Germany) at 450 nm excitation and 570 nm emission wavelengths. BDNF concentration was calculated from the standard curve generated from human recombinant BDNF provided by the manufacturer. The mean values of measurements were analyzed by a nonlinear regression fit algorithm using GraphPad Prism version 4.0 (GraphPad Software, San Diego, CA).

### 2.5 Study neuropsychological assessment

#### 2.5.1 Cambridge neuropsychological test automated battery (CANTAB)

The CANTAB battery test was used at the first, second, and third visits during the study to measure the efficacy of Colostrinin^®^ in the context of cognitive functioning among a study population. CANTAB was created at the University of Cambridge. It incorporates very sensitive, precise, and objective measures of cognitive performance that are associated with brain networks. To assess cognitive functions, mainly memory and attention, four tests selected from the CANTAB battery were offered for all three study groups (Barnett et al., 2016).

At the first visit, all participants in the MT study group completed the Multitasking Media Questionnaire (MMQ), based on which the MMI index was calculated after completion of the study. The ADT group participants at the first visit completed the Adult ADHD Self‐Report Scale Symptom Checklist (ASRS‐v1.1) to enroll into the study. AAMI/OLD group at first and last visit completed the MoCA (Montreal Cognitive Assessment) questionnaire to assess for MCI (Mild Cognitive Impairment). The cut‐off point before inclusion in the study was 26 points. The difference in MoCA scores between the first and last visit was also included in the AAMI group's efficacy assessment.

Cognitive performance index (CPI) is a composite index based on four cognitive tests taken from CANTAB neuropsychological battery. These four tests chosen carefully were then applied to all participants enrolled to the study. This empirical index will be calculated after completion of the study by all participants and it's assumed that its mean values will be different in each study group due to the demographic and environmental factors. Rationale for building such index is to establish reference value of cognitive performance for each group in the study because of lack any specific normative data regarding Polish population. The calculation of index assumes it will use proportionally weighted average of the scores each of these four tests, and finally, CPI index will be based on weighted values from following tests: Rapid visual information processing (RVP), that is a sensitive measure of the ability to maintain concentration (Gau & Huang, 2014; Hilti et al., 2010); delayed match to sample (DMS), that was created to identify perceptual competency from online information maintenance during delays, as well as to broaden prior results of working memory deficiencies in schizophrenia patients in multiple ways, by looking at working memory for visual pattern information (Lencz et al., 2003; Matthews et al., 2012); paired associates learning (PAL), that is used in general care to triage patients with memory problems, as well as in fundamental research in traditional neuropsychology and drug testing for Alzheimer's and other CNS illnesses (Barnett et al., 2016; Shimamura & Squire, 1984); reaction time (RTI) simple and choice—reaction time (SRT and CRT) is a basic motor response to a sensory input (Pirozzolo et al., 1981). For mild cognitive impairment (MCI), determination was used Montreal Cognitive Assessment (MoCA) test (Freitas et al., 2013; Nasreddine et al., 2005; Wong et al., 2015). Detailed information about particular cognitive tests are presented within Supplementary Material S1.

### 2.6 Statistical analysis

All variables were summarized using descriptive statistics. The Shapiro–Wilk test was used to test normality. The differences between samples with the normal distribution were evaluated with the Student's t‐test. For those without normal distribution, Mann‐Whitney U test was used. GraphPad Prism software v. 5 (GraphPad Software Inc.). The significance level was set at *p* < .5.

## 3 RESULTS

Volunteers involved to the study in equal proportion of men and women. Participants were divided into three groups with followed distribution of age: participants of MT group with the mean age 21.4 and range between 19 and 24 years, ADT group with mean age 38.9 and range between 25 and 54 years, and AAMI group with mean age 61.6 and range 55–74 years. Detailed distribution and age statistics are presented in Table 2.

**TABLE 2:** Age statistics within groups

Study group	Min–max	Mean	Mode	Median	SD	Standard error	Quartiles
MT	19–24	21.4	19	21.5	1.81	0.23	Q_1_ → 19.5 Q_2_ → 21.5 Q_3_ → 23
ADT	25–54	38.9	26, 28, 38, 39	39	8.91	1.17	Q_1_ → 30 Q_2_ → 39 Q_3_ → 46
OLD	55–74	61.6	57	60	5.19	0.67	Q_1_ → 57 Q_2_ → 60 Q_3_ → 64.5

### 3.1 Demographics

79% of the study participants had a high school education and 21% had a college education; 3% of study participants lived in rural areas, one person lived in a small town, 97% lived in a large city (with a population over 50,000); 38% participants in the study had a permanent job, 6% participants had own businesses, 5.6% participants were unemployed and economically inactive, 31% participants had student status, and 29% participants were economically inactive due to health or age. Half of the study participants had children. 68% of participants declared regular physical activity.

### 3.2 MoCA score before and after treatment

**FIGURE 1:**
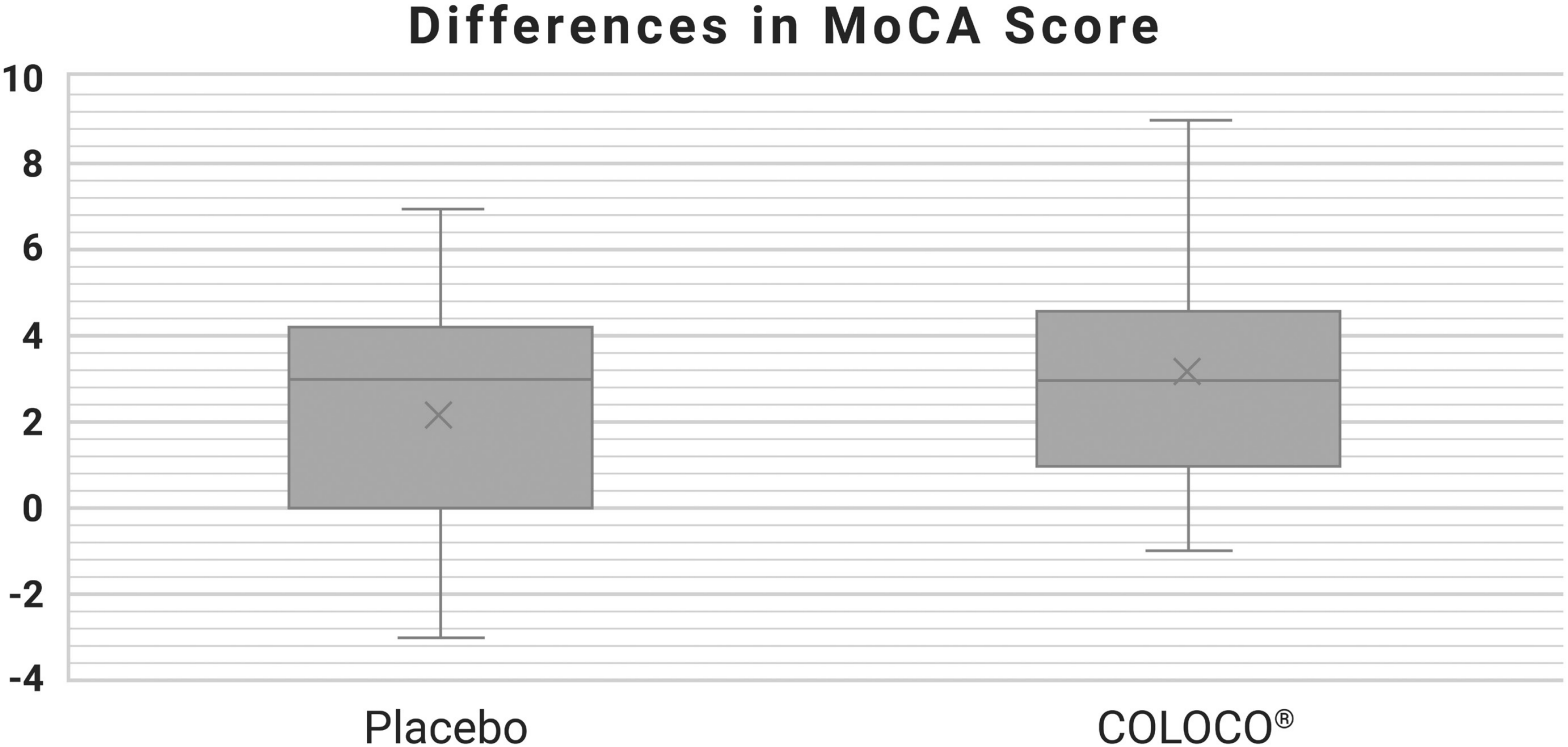
The graph presents changes in MoCA test scores in the AAMI group (*t*‐value = 2.41673, *p* < .05)

There were some differences between the average MoCA score before and after received treatment within both groups. For COLOCO^®^ group, the average difference between the MoCA test was 3.13, respectively; for the placebo group, average was 2.13 (*p* < 0.05, *t*‐value = 2.41673). The distribution of the differences between MoCA scores is presented in Figure 1.

Moreover, nine patients aged 55+ from the center in Warsaw (AAMI group) had low MoCA score below 20 at the screening visit indicating significant cognitive deficits that could indicate early stages of dementia (MCI = mild cognitive impairment). After taking COLOCO^®^ for 4 months, these subjects were retested. Compared to the other study participants assigned to the AAMI/OLD group and taking Colostrinin^®^, these nine subjects had the highest degree of cognitive improvement. In this group, the average improvement was five points (in some, it was as high as nine points), compared to 3.13 points for whole AAMI group. The small amount of these individuals was too small subgroup to obtain statistical significance for these results.

### 3.3 Circulating serum BDNF

**FIGURE 2:**
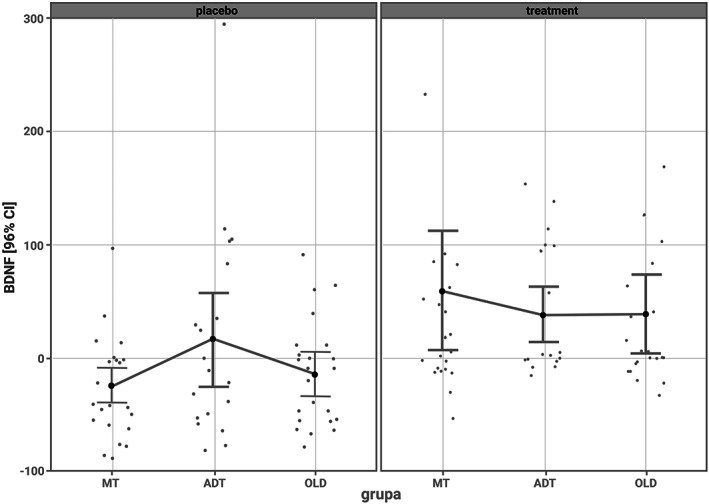
Results are presented as a mean value and standard deviation with the 95% confidence intervals in the respective age groups along with the level of significance. Serum BDNF levels of subjects in each age group (MT—age ≥18 and ≤25 years; ADT—age >25 and ≤55 years; OLD—age >55 and ≤75 years).

Circulating serum BDNF concentrations was compared between subjects treated with COLOCO^®^ and placebo controls within the groups. Considering the age groups, in the MT, ADT, and OLD group treated with Colostrinin^®^, there was an increase of serum BDNF level in 78%, 44%, and 62.5% of subjects, respectively (Figure 2). In placebo group, the level of BDNF was found higher in 18% of MT subjects, 23.5% of ADT subjects, and 30% of OLD individuals. What is interesting, biochemical analysis revealed a significant drop in BDNF concentration in particular participants. Serum BDNF level was reduced in 36.9% of subjects treated with COLOCO^®^ and in 75.8% of participants in the control group. Altogether, biochemical analysis revealed an increase of BDNF level in 63.1% of all participants treated with Colostrinin^®^ (Chi^2^ = 19.46; *p* < .001: OR = 0.1868, 95%CI = 0.0866–0.4031) in comparison to placebo‐treated group.

### 3.4 Delayed match to sample test (DMS)

**FIGURE 3:**
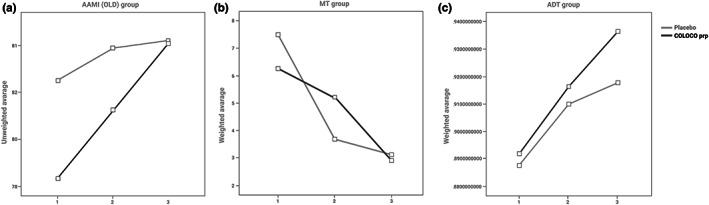
(a) The graph shows the difference in the change in DMS values in the within‐group measurement during study period for the AAMI group. In the placebo group, the mean at first visit is 82.5 (SD = 1.483), and in the Colostrinin^®^ group, it is 78.333 (SD = 1.508). At third visit, these values for the placebo group are 84.194 (SD = 1.359), respectively, while in COLOCO^®^ group, mean = 84.083 (SD = 1.381). (b) The graph shows the difference in PAL values. Total errors (adjusted) in the within‐group measurements between first, second, and third visit for the MT group (*p* < .01). (c) The graph shows the difference in the change in RVP values in the within‐group measurement system between visits for the ADT group. In the placebo group, the mean at first visit is 0.888 (SD = 0.006), and in the Colostrinin^®^ group 0.892 (SD = 0.006). At third visit, these values for the placebo group are 0.918 (SD = 0.007), for the COLOCO^®^ group 0.936 (SD = 0.007), respectively (visit 1 vs. visit 3, *p* = .084).

Among the results obtained for ADT and MT groups, no statistical significance was obtained for differences between COLOCO^®^ and control subgroups in DMS test. At a significance level p < .1, there were differences within AAMI group (F = 3.005, ε^2^ = 0.024), Figure 3a.

### 3.5 Paired associates learning (PAL)

Among the results obtained for ADT and AAMI groups in within‐subjects effects of PAL scores, there were no statistical significance for differences between Colostrinin^®^ and control subgroups. At a significance level *p* < .01, there were differences within MT group, F = 5.072, ε^2^ = 0.043, Figure 3b.

### 3.6 Reaction time (RTI) simple reaction time

Among the results obtained for MT and AAMI groups in between‐subjects effects comparison, there were no statistical significance for differences between COLOCO^®^ and control subgroups in RTI simple reaction time scores. At a significance level *p* < .1, there were differences within ADT group (*F* = 2.793, *ε* ^2^ = 0.024).

### 3.7 Rapid visual information processing (RVP)

Among the results obtained for MT and AAMI groups in within‐subjects contrast, there were no statistical significance for differences between Colostrinin^®^ and control subgroups in RTI simple reaction time scores. At a significance level *p* < .1, there were differences within ADT group, F = 3.039, ε^2^ = 0.026; Figure 3c.

### 3.8 Adverse events of COLOCO ^®^ (Colostrinin^®^)

The side effects of COLOCO^®^ were additionally examined. During the final visit ending the study, all participants were asked about adverse events occurrence during the whole period of the study last. None of patients who received Colostrinin^®^ reported adverse effects.

## 4 DISCUSSION

This was the first and pioneer study to determine the relationship between Colostrinin^®^ (COLOCO^®^) and the BDNF level in order to prove its ability to modulate cognitive skills. We receive promising results from the study regarding biochemical parameter (BDNF serum level) that confirms our thesis that PRP have the ability of cognitive skills modulation.

We examined serum (not plasma) BDNF levels because this is the most commonly used method to examine the association of blood growth factors in humans with individual differences in cognitive functioning. This way of BDNF level determination was chosen because BDNF content in serum during aging correlates with the cognitive decline, and this effect was not previously observed in reference to plasma or platelets (Lommatzsch et al., 2005). BDNF is involved in many processes related to normal functioning of the nervous system, including synaptic plasticity, long‐term memory, and maintenance of nerve cell viability (neuroprotection), and was selected to assess the efficiency of cognitive processes.

The results of our study are compliant with other studies, where the blood BDNF level decreases with the respect to the progression of age (Tapia‐Arancibia et al., 2008). The most noticeable effect of COLOCO^®^ on serum BDNF level was thereby recorded in participants over 55 years of age in whom the baseline concentration of circulating BDNF was reduced the most. In contrary, analysis of BDNF concentration in the blood samples obtained from participants in early and middle adulthood suggest a little effect of Colostrinin^®^ on BDNF concentration in healthy adults. These data indicate that COLOCO^®^ modulates the BDNF level in the specific manner to obtain the optimal effect under conditions of BDNF depletion. Current therapies designed to treat neurodegenerative disorders often demonstrate the limited efficiency; therefore, the main focus of the present study was to evaluate beneficial effects of Colostrinin^®^ on BDNF level which is crucial for proper brain functioning in healthy individuals. Lee et al. noticed that BDNF level may be depleted in patients with depression (Lee et al., 2007). Vanicek et al. noticed that electrostimulations may elevate the BDNF level in patients with depression (Vanicek et al., 2019) while our study demonstrates that there is no need to use such a disturbing methods. Marquez and colleagues prove that physical exercises (high‐intensity interval training) may also elevate the BDNF concentration (Saucedo Marquez et al., 2015); however, this way of improving BDNF level may be very difficult for elderly patients. Regarding Altar publication, our substance may be a promising agent also in depression treatment (Altar, 1999). However, in some cases, we observed no change or even decrease in BDNF concentration. This difference may be explained by the strong dependence of BDNF level on multiple factors such sex hormones (estrogens), smoking, age, physical activity, or body mass index (Begliuomini et al., 2007; Jamal et al., 2014; Lommatzsch et al., 2005).

However, it was observed that in some cases, 36.9% of subjects treated with COLOCO^®^, and in 75.8% of participants in the control group, we have noticed significant drop of BDNF concentration. As BDNF concentration can depend on many factors, it was not surprising that it can vary a lot within control group (even regarding significant drop). However, it was not expected within study group. This may indicate that Colostrinin^®^ does not act in one, straightforward manner elevating biochemical marker. This rather suggests that it may help reach some kind of desired (optimal) BDNF concentration for better brain functioning and helps maintaining cognitive skills. To explain this phenomenon, further research is necessary with larger and more homologous study group.

The Cambridge Neuropsychological Test Automated Battery (CANTAB) is a computerized test battery that covers several cognitive domains, such as induction, visual memory, executive function, attention, and semantic memory (Fray & Robbins, 1996). Not all results collected during the study had adequate statistical significance from the CANTAB test battery. Improved scores within the delayed match to sample test suggest improvements in forced choice recognition memory and short‐term visual memory. Although the results cannot be translated directly into improvements in brain function, Lencz et al. suggest possible improvements in brain structure in the temporal and frontal lobes (Lencz et al., 2003). PAL is used to detect memory problems (Barnett et al., 2016; Shimamura & Squire, 1984). Our results indicate that COLOCO^®^ improved ability to learn, retain knowledge, and recall, which may suggest improved functioning of hippocampal regions and integrity of the entorhinal and transentorhinal cortex. Colostrinin^®^ improved visual memory and learning ability in healthy volunteers. Reaction time assesses the ability, accuracy, and speed of a motor response to a sensory stimulus. Pirozollo shows that RT scores are also associated with overall brain and nervous system health, physical activity, head trauma and injury, or simply cognitive engagement (Pirozzolo et al., 1981). Our results indicate that COLOCO^®^ improved stimulus responsiveness in patients aged 25–54, which may suggest an overall improvement in brain health and function. RVP is a sensitive measure of ability and maintaining concentration. An improvement in RVP score indicates an improvement in concentration skills and suggests an improvement in overall cognitive ability (Gau & Huang, 2014; Hilti et al., 2010). Our study showed an increase in RVP in patients between the ages of 25 and 54 years under the influence of Colostrinin^®^. This suggests that COLOCO^®^ improves brain functioning in the parietal and frontal lobe areas of the brain.

Regarding MoCA score, those were the most promising results. Nasreddine and collaborates declared that MoCA is a study with very high reliability and has high sensitivity and specificity for detecting cognitive impairment—including mild cognitive impairment (Nasreddine et al., 2005). Our results showed an increase in the absolute value for MoCA scores. This may suggest a procognitive effect of colostrinin in patients with cognitive deficits. This study shows that Colostrinin^®^ improve visual–spatial skills, attention, concentration, and short‐term memory in healthy subjects over 55 years. This is very promising for elderly with dementia, Alzheimer's Disease, or patients with a history of stroke (Koski, 2013).

No adverse events were observed In any of the subjects taking COLOCO^®^. In addition, during the study, none of the participants reported any abnormal worrying symptom that could indicate an adverse event of Colostrinin^®^ usage. Being 100% natural, nonsynthetic substance with confirmed no adverse side effects, it is evident that application of COLOCO^®^ is safe and well tolerated by humans.

## 5 CONCLUSIONS

In conclusion, COLOCO^®^ has a beneficial effect in the population of healthy people on increasing the efficiency of cognitive processes crucial for cognitive homeostasis at every stage of adult life. In the youngest group, it was the ability to learn, in the middle group the ability to concentrate (attention) and the speed of reaction in response to a stimulus, while in the oldest group, it improved the efficiency of memory processes. The presented study results have evidenced that Colostrinin^®^ boosts the blood BDNF level which confirms the enhancement of cognitive results and has no adverse events in population of healthy adults.

## CONFLICT OF INTEREST

The scientific study was conducted by MTZ Clinical Research Sp. z o. o. on behalf of Geo‐Poland Sp. z o. o. according to protocol CO/GP/08/18. The study was financed by Geo‐Poland sp. z o. o. Magdalena Kuszczyk is co‐owner of a patent for the use of proline‐rich proteins: High‐proline peptide complex for applications in the prophylaxis and treatment support of disorders and morbidities related to changes in the neurotrophic factor of brain origin, and for modulating it (Dobrzyński & Bendarek, 2020).

## Supporting information


Data S1

## Data Availability

Research data are not shared.
