# Supplementary material for: Proline‐rich polypeptides (Colostrinin®/COLOCO ®) modulate BDNF concentration in blood affecting cognitive function in adults: A double‐blind randomized placebo‐controlled study
Source: Food Sci Nutr. 2023 Jan 11;11(3):1477–85. doi: 10.1002/fsn3.3187 (PMC10002942; doi:10.1002/fsn3.3187)
Supplement: Supplementary file 1 — Data S1 [file FSN3-11-1477-s001.docx]

Supplementary material 1

- Rapid Visual Information Processing (RVP)

RVP is a sensitive measure of the ability to maintain concentration. The higher the score the better. Th e RVP test, which measures sustained attention, is used in behavioural research and has increasingly been employed in neuroimaging investigations. It is a test of sustained attention and has been proved to be useful in many studies in which drugs are used to help develop a disease or dysfunction model. It is sensitive to dysfunction in the parietal and frontal lobe areas of the brain and is also a sensitive measure of general performance. [23,24]

Task: A white box appears in the centre of the computer screen, inside which digits from 2 to 9 appears in pseudo-random order, at the rate of 100 digits per minute. Participants are requested to detect target sequences of digits and to register responses using the press pad.

- **Delayed Match to Sample (DMS)**

The test was created to identify perceptual competency from on-line information maintenance during delays, as well as to broaden prior results of working memory deficiencies in schizophrenia patients in multiple ways, by looking at working memory for visual pattern information. Participants were presented a complicated visual pattern and then had to choose the exact same pattern from four different answer options. The task's difficulty was adjusted by varying the time between the display of test stimulus and the four response stimuli (0, 4, to 12 s) [25, 26]. This test assesses forced choice recognition memory for novel non-verbalised patterns and tests both simultaneous and short-term visual memory. This test is primarily sensitive to damage in the medical temporal lobe area with some input from the frontal lobes.

Task: The participant is shown a complex visual pattern and then after a brief delay, four similar patterns. The participants must touch the pattern which exactly matches the sample. In both cases higher score is better.

- **Paired Associates Learning (PAL)**

PAL is used in general care to triage patients with memory problems, as well as in fundamental research in traditional neuropsychology and drug testing for Alzheimer's and other CNS illnesses. The capacity to acquire, preserve, and recall knowledge about prior events is known as episodic memory. In animals episodic memory can be defined by 'what, when, and where' whereas in humans, infrequently relies on remembering all three. PAL is a series of trials in which the subject learns where one or more visual patterns on the screen are located. Proper functioning of the hippocampus regions, as well as the integrity of the entorhinal and transentorhinal cortex, are critical for this 'object-location memory'. The exam becomes more difficult as it progresses; at the beginning, there is just one pattern to remember, and at the end, there are eight patterns to recall. PAL assesses visual memory and learning ability. It is a key tool for evaluating subjects with potential dementia disorders, Alzheimer's disease, or age-related memory loss. [22, 27] The lower the score the better.

This challenging test assesses visual memory and new learning and is a useful tool for assessing individuals with questionable dementia, mild cognitive impairment, Alzheimer’s disease and age-related memory loss.

Task: boxes are displayed on the screen and are opened in a randomised order. One or more of them will contain a pattern. The patterns are then displayed in the middle of the screen, one at a time and the participant must touch the box, where the patter was originally located. If the participant makes an error the patterns are represented to remind the participant of their locations. The difficulty level increases through the test. In the clinical mode, the number of patterns increases from one to eight, which challenges even very able participants.

- **Reaction Time (RTI) Simple and Choice – Reaction Time (SRT and CRT)**

Reaction time is a basic motor response to a sensory input. RTI examines motor and mental speed of response to a stimulus, as well as reaction time, accuracy of response to a stimulus, and impulsivity. Since the mid-nineteenth century RTI has been assumed to represent the speed of nerve conduction. RTI is sensitive to a wide range of subject and external factors. For example aging causes all volitional motions to slow down. [18] Many studies underline the fact, that the RT results are connected also with brain and nervous system health in general, physical activity, head injuries and trauma or simply by cognitive involvement. [28] The lower the score the better. RTI is a latency task with a comparative history and uses a procedure to separate response latency from movement time. It is more useful than CRT or SRT where it is necessary to control for tremor.

Task: the task is divided into five stages, which require increasingly complex chains of responses. In each case, the participant must react as soon as a yellow dot appears. In some stages the dot may appear in one of five locations and the participant must sometimes respond by using the press-pad sometimes by touching the screen and sometimes both.

- **Montreal Cognitive Assessment (MoCA)**

The MoCA is a quick and easy test for mild cognitive impairment (MCI) [29–31]. The MoCA showed high sensitivity and specificity for diagnosing MCI in a validation study, as well as strong test-retest reliability and positive and negative predictive values for MCI and Alzheimer's disease [29]. It's a short (10 minutes), single page, 30-point test. The short-term memory recall task consists of two learning trials of 5 nouns followed by a 5-minute delay in recall. A clock-drawing activity and a 3D cube copy are used to measure visuospatial abilities. An alternation task derived from the Trail Making B task, a phonemic fluency task, and a 2-item verbal abstraction task are used to measure multiple areas of executive processes. A sustained attention task, a serial subtraction problem, and digits forward and backward are used to assess attention, concentration, and working memory. A three-item confrontation naming task with low-familiarity animals, repetition of two syntactically complex sentences, and the fluency task are used to assess language. The general orientation (to time and place) also was assessed [29].
